# Which Groups of Children Are at More Risk of Fatality during COVID-19 Pandemic? A Case-Control Study in Yazd, Iran

**DOI:** 10.1155/2023/8838056

**Published:** 2023-12-14

**Authors:** Behnam Shafaei, Zahra Nafei, Mehran Karimi, Nasrin Behniafard, Farimah Shamsi, Masoud Faisal, Amir Pasha Amel Shahbaz, Elahe Akbarian

**Affiliations:** ^1^Children Growth Disorder Research Center, Shahid Sadoughi University of Medical Sciences, Yazd, Iran; ^2^Center of Healthcare Data Modeling, Department of Biostatics and Epidemiology, School of Public Health, Shahid Sadoughi University of Sciences, Yazd, Iran; ^3^Department of Radiology, Faculty of Medicine, Shahid Sadoughi University of Medical Sciences, Yazd, Iran

## Abstract

**Introduction:**

The study aims to investigate the characteristics, comorbidities, laboratory findings, and clinical manifestations of under 18-year-old patients who died with the diagnosis of COVID-19 and determination of the most prevalent risk factors.

**Method:**

This case-control study was performed at a referral hospital in Yazd from March 2020 to August 2021. All patients under 18 years who were diagnosed through real-time RT-PCR, chest computed tomography, and the World Health Organization definition were divided into deceased and survived groups. The characteristics (age and sex), disease severity, comorbidities, laboratory findings, and clinical manifestations of the two groups were compared and analyzed using SPSS, version 18 (SPSS Inc., Chicago, III., USA).

**Results:**

A total of 24 patients in the deceased group and 167 patients in the survived group were compared. The highest mortality rate was observed in the age group of 1 month to 5 years, although no statistically significant relationship was found between age groups and the risk of mortality. Disease severity, dyspnea, low oxygen saturation on admission, length of hospital stays, and hospitalization history before the last admission were significantly correlated with mortality (*P* < 0.05). Lymphopenia increased the probability of mortality by more than two times (OR: 2.568; 95% CI (0.962–6.852)), but this was not the case for D-dimer and C-reactive protein. Furthermore, 27.5% of survived patients had normal chest CT scans, which was a statistically significant difference compared to the deceased patients (*P*: 0.031).

**Conclusion:**

Based on the findings of this study, dyspnea, low oxygen saturation, and lymphopenia are critical indicators for identifying high-risk children with COVID-19 and triaging them for better care and treatment.

## 1. Introduction

Even though more than three years have passed since COVID-19 was declared a pandemic and resulted in the death of more than six million people worldwide, there are still many unanswered questions about this disease [1]. Previous studies have revealed that children are less susceptible to COVID-19 and experience milder symptoms compared to adults. Furthermore, more recent investigations have indicated that the severity of the disease in children during the second and third waves of the pandemic is either comparable to or even lower than what was observed during the first wave [2–7].

The most frequently observed clinical symptoms in children with COVID-19 include fever, cough, and sore throat. As the disease progresses, there is a possibility of experiencing dyspnea, which can eventually lead to the development of acute respiratory distress syndrome (ARDS) or multiorgan failure [8]. Additionally, lymphopenia, increased serum D-dimer, and procalcitonin levels are prevalent laboratory findings. Chest computed tomography (CT) scans often reveal patchy lesions and ground-glass opacities [9, 10]. Certain factors, such as male gender, underlying diseases, age under one month, and symptoms of lower respiratory tract infection, increase the risk of fatal conditions and death [11, 12].

Studies conducted in the United States of America and Europe have identified a distinct medical condition known as pediatric inflammatory multisystem syndrome temporally associated with SARS-CoV-2 (PIMS-TS) or multisystem inflammatory syndrome in children (MIS-C). This condition has been found to be closely associated with the severity of COVID-19 [13, 14]. The symptoms of PIMS-TS or MIS-C closely resemble those of Kawasaki disease (KD). They exhibit characteristics such as cytokine storms, macrophage activation, hyper-inflammatory traits, and elevated levels of proinflammatory cytokines such as IL-1, IL-6, and TNF-*α* [15]. Patients with MIS-C often present with gastrointestinal, mucocutaneous, and cardiovascular symptoms, and the condition is associated with higher disease severity [16, 17].

The progression of COVID-19 typically includes unpredictable events, beginning with an asymptomatic incubation period that can persist for several days, followed by a sudden onset of respiratory symptoms, which can eventually lead to severe stages requiring hospitalization in an intensive care unit and mechanical ventilation. Throughout infection, the body's immune and inflammatory responses can be divided into three distinct phases: an initial innate immune response focused on the lungs, followed by a local/systemic immune response, and potentially progressing to excessive inflammatory responses that may trigger a cytokine storm and result in ARDS and multiple organ failure. Cytokine storm-like syndromes have the potential to induce a series of abnormalities and tissue damage, encompassing lymphocyte dysfunction, irregularities in granulocytes/monocytes, damage to the vascular barrier, capillary impairment, diffuse alveolar damage, multiorgan failure, and even fatal outcomes. In other words, the cytokine storm induced by the virus correlates with the severity of COVID-19 [8, 15]. On the other hand, research has indicated that patients with primary immune system deficiencies, particularly those with agammaglobulinemia and the absence of B lymphocytes, tend to experience a milder course of COVID-19 with more favorable outcomes. However, in patients with common variable immunodeficiency (CVID) and dysfunctional B lymphocytes, the disease often takes a severe form, necessitating ICU admission, mechanical ventilation, and treatment with IL-6-blocking drugs [18].

The objective of this study was to explore the characteristics, comorbidities, laboratory findings, and clinical manifestations of patients under 18 years old who died from COVID-19. Additionally, we aimed to identify the most prevalent risk factors associated with mortality in this population.

## 2. Methods

### 2.1. Study Design and Data Collection

This case-control study enrolled all patients under 18 years old who were admitted to a referral hospital in Yazd and diagnosed with COVID-19 between March 2020 and August 2021. The study utilized a census sampling method, which did not exclude any patients from the research. All data were extracted from the patients' archived medical records at the hospital. The patients were categorized into two distinct groups: the deceased group, consisting of patients who died during their hospitalization, and the survived group, comprising those who were discharged. Patient categorization as confirmed, probable, and suspected cases was determined according to the criteria defined by the World Health Organization (WHO) [19].

We conducted a comparative analysis of various factors, including demographic variables (age and sex), disease severity, underlying medical conditions, clinical presentations, laboratory results, hospitalization duration, pediatric intensive care unit (PICU) admission, reverse transcription polymerase chain reaction (RT-PCR) test outcomes, and spiral chest CT scan findings (pattern and severity score) between the two study groups. Disease severity was categorized into three distinct levels: nonsevere, severe, and critical, in accordance with WHO guidelines [20]. According to the World Health Organization (WHO) criteria, patients were categorized into three groups: confirmed (positive PCR regardless of symptoms), probable (positive lung CT findings in patients with negative or unknown PCR results), and suspected (children with a history of respiratory symptoms, with or without fever, and a documented close contact with a probable or confirmed case of COVID-19).

To diagnose confirmed cases of COVID-19, we employed the Novel Coronavirus (2019-nCoV) Nucleic Acid Diagnostic Kit (PCR-Fluorescence Probing) developed by the Sansure Biotech Company. This kit is used for the qualitative detection of the ORF1ab and N genes of SARS-CoV-2 RNA, and a cycle threshold (Ct) ≤ 40 was considered positive. All the results were interpreted by a trained technician based on positive and negative controls. Furthermore, the test exhibited a sensitivity of 95%.

A D-dimer level above 200 mg/dl and a C-reactive protein (CRP) level above 10 mg/dl were considered positive. The definition of lymphopenia differed in different age groups of children, and we assessed it individually.

The pattern and severity of chest CT scans were evaluated by an expert radiologist. The severity scoring system was based on the visual assessment of each lobe's involvement. The chest CT score for each of the five lobes was calculated according to the extent of anatomical involvement (scoring from 1 to 5), and the overall severity of the five lobes demonstrated as mild (≤7), moderate (8–17), and severe (≥18) [21].

The ethics committee of Shahid Sadoughi University of Medical Sciences, Yazd, Iran, approved this study (ethical code: IR.SSU.MEDICINE.REC.1401.027).

### 2.2. Statistical Analysis

The data were analyzed using descriptive and dispersion statistics to report the results. The chi-square test and Fisher exact test were employed to compare the frequency of explanatory variables between the survived and deceased patients. Logistic regression was conducted to estimate the risk of death among deceased patients based on the groups of explanatory variables, with comparison to the survived children. The statistical software SPSS Statistics for Windows, version 18 (SPSS Inc., Chicago, III., USA) was used for data analysis. A significance level of 0.05 was employed for all tests, and all statistical analyses were conducted with a two-tailed approach.

## 3. Results

### 3.1. Demographic Characteristics and Disease Severity

Out of these patients, 133 (69.6%) were confirmed cases, 26 (13.6%) were considered probable cases, and 32 (16.8%) were suspected cases. The deceased and survived groups included 24 and 167 patients, respectively. The PCR test was positive in 62.5% of the deceased group and 70.7% of the survived group (*P* = 0.416). The demographic characteristics and disease severity of the 191 patients under 18 years old are presented in Table 1.

### 3.2. Underlying Disease

In the group of deceased patients, it was found that 15 individuals (62.5%) had preexisting health conditions, with malignancy and neurologic disease being the most frequently observed underlying diseases, respectively (Table 2).

### 3.3. Signs and Symptoms

In our study, fever emerged as the most prevalent symptom in both the deceased and survival groups in on-admission and preadmission time. Dyspnea was more common in the deceased group with a significant difference (Table 3).

### 3.4. Laboratory Findings

A comparison of laboratory findings on the first day of admission was conducted for both groups, and no significant differences were observed (Table 4).

Lymphopenia was observed in 47.4% of patients in the deceased group and 26.4% of patients in the survived group (**P** **=** **0.054**) (OR = 2.568, 95% CI (0.962–6.852)).

Among patients whose oxygen saturation (O_2_ sat) was recorded on admission, 57.1% of the deceased group and 29.6% of the surviving group had O_2_ sat <94% (**P** **=** **0.037**) (OR = 3.171, 95% CI: (1.028–9.778)).

D-dimer and C-reactive protein (CRP) levels were measured in two groups, revealing no significant difference between them.

### 3.5. Chest CT Scan Pattern and Severity

In our study, we evaluated the pattern and severity score of chest spiral CT scans in 82 patients. The results showed that ground-glass opacity and consolidation were the predominant findings, as summarized in Table 5.

### 3.6. Other Findings

A significant difference in the length of hospital stay was observed between the two groups. Specifically, the percentage of patients who were hospitalized for less than 7 days was 78.4% in the group that survived, while it was 50% in the deceased group (**P** **=** **0.011**).

Additionally, the history of hospitalization due to COVID-19 before admission was significantly higher among deceased patients compared to the survived group. The percentage of patients with previous COVID-19 hospitalizations was 50% in the deceased group and 22% in the survived group (50% vs. 22%) (**P** **=** **0.001**) (OR = 4.252, 95% CI: (1.661–10.884)).

As expected, the number of patients admitted to the pediatric intensive care unit (PICU) was significantly higher in the deceased group compared to the survived group (**P** **<** **0.001**) (OR = 37.342, 95% CI: (8.399–166.030)).

Furthermore, a higher proportion of patients in the deceased group (37.5%) fulfilled the criteria for the multisystem inflammatory syndrome in children (MIS-C) compared to the survived group (21.6%), although this difference was not statistically significant (*P* = 0.085) (OR = 2.183, 95% CI: (0.883–5.397)).

## 4. Discussion

### 4.1. Statistically Significant Findings

In this study, we conducted a comparison between the characteristics, comorbidities, laboratory findings, and clinical manifestations of 24 patients under 18 years old who died and 167 patients who survived COVID-19. Our findings revealed several significant factors that increased the risk of death. These factors included disease severity, presence of dyspnea, lymphopenia, hospital stay of more than 7 days, previous hospitalization before admission to our center, and low oxygen saturation levels. Additionally, we observed that the survived group had a higher prevalence of normal chest CT scan findings. In our study, consistent with other similar studies, ground glass opacity (GGO) was found to be the most common finding in lung CT scans of pediatric COVID-19 cases. However, no significant correlation was found between GGO and mortality. Instead, our findings highlight the importance of considering other indicators, such as oxygen saturation and dyspnea upon admission, for a more comprehensive assessment of outcomes. It is possible that a larger study with a greater sample size could potentially yield different or contrasting results.

### 4.2. Disease Severity Was Highly Correlated, but Age and Gender Were Not

As expected, the severity of the disease showed a significant correlation with the risk of mortality in the children we studied. A recent study conducted in Iran, involving 47 hospitalized COVID-19 patients, found that symptoms such as dyspnea, vomiting, and chest pain were significantly associated with disease severity. Some of the findings of their study are consistent with the results of our study [22].

Furthermore, our study identified a higher frequency of deaths within the 1 month to 5 years age group compared to other age groups. However, it is important to note that no statistically significant relationship was observed between any specific age group and the risk of mortality. However, some studies suggest a higher frequency of deaths among younger age groups compared to older ones [23, 24].

Additionally, a systematic review of 83 studies did not find any association between gender and death in children. However, they did identify an increased risk of death in patients aged 1 to 4 years and infants [25]. Consistent with other studies, our study also found that male patients were the majority in the deceased group [11, 26].

Furthermore, our study revealed a significant correlation between previous hospitalization due to COVID-19 and increased mortality (*P* < 0.001). The reason behind this finding can be attributed to the fact that our center is specifically designated as a referral hospital for COVID-19 cases in our region, and critically ill patients who need special care and hospitalization in the PICU were referred to this center.

### 4.3. Dyspnea and Low Oxygen Saturation Raised the Risk of Death, and Fever Was the Most Common

Our study confirmed that dyspnea on admission time was strongly correlated with mortality, consistent with a study conducted in Vietnam [27]. Fever was the most prevalent symptom observed in both the deceased and survived groups, followed by vomiting and dyspnea in the deceased group, and cough in the survived group, aligning with previous research [28–34]. Notably, despite the high frequency of fever and vomiting among the deceased group, there was no statistically significant increase in the risk of death, which corresponds with findings from a study conducted in Brazil [35]. Several studies have identified low oxygen saturation as a frequent occurrence among children infected with COVID-19. Additionally, our study findings support the existence of a significant association between this issue and mortality rates [10, 35, 36]. Since, in some studies, dyspnea and low O_2_ saturation are related to the COVID-19 disease severity as an independent factor, it can justify the significant relationship between shortness of breath and mortality [27, 35]. Low O_2_ saturation and dyspnea in children with COVID-19 can indicate the severity of lung involvement, the continuation of which leads to damage to vital organs and increases the chance of death.

### 4.4. The Pattern and Severity of Chest CT Scan Had No Significant Relationship with Death

Our study showed that none of the children who died due to COVID-19 had a normal CT scan, which could be due to the impact of lung involvement on the patient's outcome. The most common pattern seen in both groups was ground glass opacity. These findings were consistent with a systematic review that included 3557 children with COVID-19 [37]. Another meta-analysis by Nino et al. also reported ground glass opacity as the most common pattern in children, which aligns with our study [38]. Consistent with our study, an abnormal chest CT scan in a study conducted in Istanbul showed a statistically significant relationship with the severity of the disease of COVID-19, which was associated with a relative increase in the risk of mortality [39].

### 4.5. Lymphopenia Was the Only Laboratory Parameter with a Statistically Significant Relationship with Mortality

This study revealed that lymphopenia was the only laboratory parameter that showed a statistically significant correlation with mortality in COVID-19 patients. This could be attributed to the uncontrolled progression of the pathogen in critically ill patients, which is often accompanied by hypercytokinemia [40]. As lymphocytes play a crucial role against viruses, lymphopenia can create a vicious cycle that ultimately exacerbates the severity of the disease. A separate cohort study conducted in Korea also indicated that lymphopenia, as well as its severity, can serve as reliable predictors of poor clinical outcomes, including mortality, the need for intensive care, and oxygen requirement [41]. Furthermore, a review study suggested that lymphopenia could be a strong predictive factor for the severe and progressive course of COVID-19 [42].

A systematic review published in October 2020 revealed that hypoalbuminemia, lymphopenia, leukocytosis, elevated levels of interleukin 6, and prolonged PT time were found to be associated with mortality in children [43].

### 4.6. No Statistically Significant Relationship Found in Underlying Disease

In this study, malignancy was the most common underlying disease in died and survived groups. However, no significant correlation was observed between the presence of underlying disease and the risk of mortality. Some studies showed that underlying respiratory and neurologic diseases were the most common underlying conditions in children who died from COVID-19 [44, 45].

## 5. Conclusion

Based on the findings of this study, dyspnea, low oxygen saturation, and lymphopenia emerged as pivotal markers for identifying high-risk children. These critical factors can serve as valuable tools for efficient triage and ensuring enhanced care and treatment for these individuals. These factors can be used for triaging purposes to ensure better care and treatment for these individuals. Also, it is necessary for the treatment staff and children's caregivers to receive more training about these indicators.

## Figures and Tables

**Table 1:** Demographic characteristics and disease severity of children with COVID-19.

Variables		Deceased (*n* = 24)	Survived (*n* = 167)	*P* value
Sex	Male	13 (54.2%)	82 (49.1%)	0.64
	Female	11 (45.8%)	85 (50.9%)	

Age groups	Under 1 month	0	8 (4.8%)	0.22
	1 month to 5 years	14 (58.3%)	73 (43.7%)	
	5 years to 13 years	4 (16.7%)	56 (33.5%)	
	13 years to 18 years	6 (25%)	30 (18%)	

Disease severity	Not severe	3 (12.5%)	94 (56.3%)	**<0.001**
	Severe	10 (41.7%)	58 (34.7%)	
	Critical	11 (45.8%)	15 (9%)	

Severe and critical diseases were observed significantly more in the deceased group.

**Table 2:** Underlying diseases in survived and deceased groups of children with COVID-19.

Variables		Deceased (*n* = 24)	Survived (*n* = 167)	*P* value	OR (95% CI) (ref: survived group)
Underlying diseases	Malignancy	5 (33.33%)	10 (14.08%)	0.75	2.229 (0.912–5.447)
	Neurologic	3 (20%)	21 (29.57%)		
	Nephrologic	2 (13.33%)	4 (5.63%)		
	Hematologic	1 (6.66%)	11 (15.49%)		
	Respiratory	1 (6.66%)	4 (5.63%)		
	Cardiovascular	1 (6.66%)	6 (8.45%)		
	Gastrointestinal	1 (6.66%)	3 (4.22%)		
	Genetic	1 (6.66%)	4 (5.63%)		
	Metabolic	0	5 (7.04%)		
	Immunologic	0	1 (1.40%)		
	Rheumatologic	0	2 (2.81%)		
	Total	**15 (100%)**	**71 (100%)**		

The odds of death in children with underlying diseases was approximately 2.23 times higher than that in children without underlying diseases.

**Table 3:** Signs and symptoms in preadmission and on-admission time in deceased and survived children with COVID-19.

Variables	Preadmission (OR ref: survived group)				On-admission (OR ref: survived group)			
	Deceased (*n* = 24)	Survived (*n* = 167)	*P*	OR (95% CI)	Deceased (*n* = 24)	Survived (*n* = 167)	*P*	OR (95% CI)
Fever	17 (70.8%)	111 (66.4%)	0.67	1.22 (0.48–3.12)	18 (75%)	104 (62.27%)	0.22	1.81 (0.68–4.82)
Vomiting	10 (41.66%)	46 (27.5%)	0.15	1.88 (0.78–4.53)	9 (37.5%)	37 (22.15%)	0.1	2.1 (0.85–5.2)
Cough	7 (29.1%)	55 (32.9%)	0.71	0.84 (0.33–2.14)	4 (16.66%)	55 (32.9%)	0.10	0.4 (0.13–1.25)
Diarrhea	6 (25%)	49 (29.34%)	0.66	0.8 (0.3–2.14)	7 (29.16%)	41 (24.5%)	0.62	1.26 (0.49–3.26)
Dyspnea	6 (25%)	46 (27.5%)	0.79	0.88 (0.33–2.34)	9 (37.5%)	33 (19.76%)	**0.05**	2.44 (0.98–6.05)
Seizure	2 (8.3%)	20 (11.97%)	0.6	0.67 (0.15–3.06)	2 (8.2%)	19 (11.37%)	0.66	0.7 (0.15–3.25)
Myalgia	1 (4.1%)	27 (16.16%)	0.12	0.22 (0.03–1.74)	1 (4.1%)	18 (10.77%)	0.48	0.36 (0.05–2.83)
Cyanosis	0	4 (2.3%)	1.00	—	1 (4.1%)	7 (4.19%)	0.99	0.99 (0.18–8.45)
Headache	0	25 (14.9%)	0.15	0.25 (0.03–1.91)	1 (4.1%)	15 (8.9%)	0.27	0.44 (0.06–3.5)

Dyspnea on admission was observed significantly more in the deceased group compared to the survived group.

**Table 4:** Laboratory findings of deceased and survived children with COVID-19.

Variables^*∗*^ (normal range)	Deceased group			Survived group			*P* value	OR (95% CI) (ref: normal range)	
	Below normal range (*n*)	Normal range (*n*)	Above normal range (*n*)	Below normal range (*n*)	Normal range (*n*)	Above normal range (*n*)			
								Lower limit range	Upper limit range
Sodium (135–145)	9 (42.9%)	11 (52.4%)	1 (4.8%)	52 (36.4%)	88 (61.5%)	3 (2.1%)	0.60	1.385 (0.538–3.563)	2.667 (0.255–27.916)
Potassium (3.5–5.5)	2 (10%)	18 (90%)	0	11 (8.0%)	121 (88.3%)	5 (3.6%)	0.66	1.222 (0.250–5.969)	0.000
Platelets count (150,000–450,000)	6 (25%)	15 (62.5%)	3 (12.5%)	29 (19%)	102 (66.7%)	22 (14.4%)	0.78	1.407 (0.501–3.951)	0.927 (0.247–3.480)
WBC count (5000–15000)	6 (26.1%)	15 (65.2%)	2 (8.7%)	31 (20%)	95 (61.3%)	29 (18.7%)	0.45	1.226 (0.438–3.433)	0.437 (0.094–2.023)
Calcium (8.8–10.8)	12 (70.6%)	5 (29.4%)	0	33 (42.9%)	43 (55.8%)	1 (1.3%)	0.11	3.127 (1.003–9.755)	0.000
Phosphorus (3–6)	2 (16.7%)	9 (75%)	1 (8.3%)	5 (11.6%)	35 (81.4%)	3 (7%)	0.87	—	—
PT (<12)	—	2 (18.2%)	9 (81.8%)	—	12 (27.3%)	32 (72.7%)	0.53	—	1.687 (0.318–8.961)
PTT (<36)	—	10 (90.9%)	1 (9.1%)	—	31 (70.5%)	13 (29.5%)	0.16	—	
ALT (<35)	—	9 (56.3%)	7 (43.8%)	—	56 (60.2%)	37 (39.8%)	0.76	—	1.177 (0.403–3.437)
AST (<45)	—	6 (37.5%)	10 (62.5%)	—	57 (60.6%)	37 (39.4%)	0.08	—	2.568 (0.860–7.663)
Magnesium (>1.5)	0	16 (100%)	—	2 (3.8%)	51 (96.2%)	—	0.43	—	—
ESR (<20)	—	7 (46.7%)	8 (53.3%)	—	45 (54.2%)	38 (45.8%)	0.59	—	1.353 (0.449–4.076)
CPK (<200)	—	6 (66.7%)	3 (33.3%)	—	61 (87.1%)	9 (12.9%)	0.10	—	—
Albumin (>3.5)	2 (40%)	3 (60%)		11 (61.1%)	7 (38.9%)	—	0.40	—	—
Blood glucose (60–200)	0	13 (92.9%)	1 (7.1%)	3 (3.4%)	81 (91%)	5 (5.6%)	0.76	—	—
LDH (<500)	—	5 (41.7%)	7 (58.3%)	—	41 (48.2%)	44 (51.8%)	0.67	—	1.305 (0.384–4.436)

^*∗*^Laboratory findings include sodium, potassium, platelets count, weight blood cell (WBC) count, calcium, phosphorus, prothrombin time (PT), partial thromboplastin time (PTT), alanine transaminase (ALT), aspartate aminotransferase (AST), magnesium, erythrocyte sedimentation rate (ESR), creatine phosphokinase (CPK), albumin, blood glucose, and lactate dehydrogenase (LDH) were assessed into two groups at the first day of admission and number of patients who had pathological laboratory marker as upper and lower limits of normal range were recorded.

**Table 5:** Pattern and severity scores of spiral chest CT scan imaging in pediatric patients with COVID-19.

Variables		Deceased	Survived	*P* value	OR (95% CI)
Pattern	Ground glass opacity	8 (61.5%)	33 (47.8%)	0.36	—
	Consolidation	6 (46.2%)	18 (26.1%)	0.14	—
	Pleural effusion	2 (15.4%)	5 (7.2%)	0.33	—
	Atelectasis	1 (7.7%)	11 (15.9%)	0.44	—
	Normal	0	19 (27.5%)	**0.031**	—

Severity	Severity score ≤ 7	3 (27.3%)	5 (16.1%)	0.36	Ref
	Severity score 8–17	4 (36.4%)	19 (61.3%)		0.351 (0.058–2.106)
	Severity score ≥ 18	4 (36.4%)	7 (22.6%)		0.952 (0.144–6.281)

Normal chest CT scan was not observed in any of the deceased children, In addition, the predominant findings of chest CT scans in both deceased and survived patients were ground glass opacity and consolidation.

## Data Availability

The registered data utilized to generate the findings of this study are subject to restrictions imposed by the Ethics Committee of Shahid Sadoughi University of Medical Sciences and Health Services, aiming to safeguard patient privacy. However, researchers who satisfy the criteria for accessing confidential data can make a data request to Mehran Karimi at drmehrankarimi@yahoo.com.
